# Skin necrosis in smoking patients receiving partial breast irradiation: two case reports

**DOI:** 10.1186/1757-1626-1-230

**Published:** 2008-10-08

**Authors:** Edward R Sauter, Steven J Westgate, Janice Templemire

**Affiliations:** 1 Departments of Surgery [ERS, JT] and Radiology [SW], Ellis Fischel Cancer Center, Columbia, MO 65212, USA; 2 Department of Surgery [ERS], University of North Dakota, Grand Forks, ND 58202, USA; 3 University of North Dakota School of Medicine and Health Sciences, 501 N. Columbia Rd, Department of Surgery, Grand Forks, ND 58202, USA

## Abstract

Partial breast irradiation has become an increasingly popular mode of treatment after excisional biopsy to treat early stage invasive breast cancer. Its main advantage is that treatment can be delivered in five days rather than 30, as is standard for whole breast irradiation. Early reports suggest good to excellent cosmesis in the vast majority of subjects. Herein we report two cases of skin necrosis in women with Stage 1 breast cancer who smoked before and after partial breast irradiation.

## Case presentation

### Case 1

48 year old Caucasian female active smoker who weighed 98 kg, ht 168 cm, with a 28 pack year smoking history underwent excisional biopsy and sentinel node dissection for a T1cN0 right breast cancer. Her only pertinent medical history was occasional dysesthesias in her upper extremity following a motor vehicle accident. A reexcision was required for one involved and two close margins. At the time of reexcision, a Mammosite^® ^partial breast irradiation catheter was inserted. The patient underwent evaluation by a radiation oncologist, who deemed the minimum cavity to skin distance to be 11 mm, as well as the conformation of the balloon within the cavity to be satisfactory for radiation delivery. The patient continued to smoke after surgery. At one month after radiation there was an eschar noted which was draining clear yellow fluid. This subsequently became infected, requiring debridement and healing of the open wound by secondary intention.

### Case 2

45 year old female active smoker who weighted 62 kg, ht 171 cm, with a 45 pack year smoking history underwent excisional biopsy and sentinel node dissection for a T1bN0 left breast cancer. Her only medical history was discomfort in her neck and back which was felt to be due to her work, which requires heavy lifting. A Mammosite partial breast irradiation catheter was inserted. The patient underwent evaluation by a radiation oncologist, who deemed the minimum cavity surface to skin distance to be 5 mm, as well as the conformation of the balloon within the cavity to be satisfactory for radiation delivery. The patient continued to smoke after surgery despite being instructed to stop. One month after surgery the wound had redness. It was unclear if this was radiation effect, infection, or both. The patient was treated with antibiotics. The redness resolved, but a 1 × 1 cm area of skin developed obvious necrosis with clear yellow drainage, requiring debridement. The wound is now healing by secondary intention.

## Discussion

Partial breast irradiation is well tolerated, with good to excellent results reported in over 80% of cases [1,2]. Skin to cavity distance appears to affect cosmesis, with some [1] but not all [2] publications reporting significantly improved cosmesis with skin spacing > = 7 mm. Complications reported include seromas, fat necrosis and infections. We are not aware of a report linking a complication from balloon-delivered partial breast irradiation to smoking. On the other hand, smoking is known to increase perioperative complications after breast implant reconstruction for cancer, in which a foreign body is placed underlying the skin after removal of the breast [3], and smokers are known to have more late normal tissue complications following whole breast radiotherapy for breast cancer [4].

## Consent

Written informed consent was obtained from the patients for publication of this care report. A copy of the written consent is available for review by the Editor-in-Chief of this journal.

## Competing interests

The authors declare that they have no competing interests.

## Authors' contributions

ES wrote the initial draft of and helped revise the manuscript. JT obtained consent from the patients and helped revise the manuscript. SW assisted with manuscript revision. All authors read and approved the final manuscript.
